# Formononetin, J1 and J2 have different effects on endothelial cells via EWSAT1‐TRAF6 and its downstream pathway

**DOI:** 10.1111/jcmm.14797

**Published:** 2019-11-19

**Authors:** Xin Li, Chen Huang, Cheng Liang Sui, Chun Mei Liang, Guang Ying Qi, Qian Yao Ren, Jian Chen, Zhao Quan Huang

**Affiliations:** ^1^ Department of Pathology and Pathophysiology Xiangya Hospital Central South University Changsha China; ^2^ Key Laboratory of Tumor Immunology and Microenvironmental Regulation Guilin Medical University Guilin China; ^3^ Epidemiology Unit Faculty of Medicine Prince of Songkla University Hat Yai Thailand

**Keywords:** EWSAT1, formononetin, HUVEC, J1, J2, TRAF6

## Abstract

Formononetin is a natural isoflavone compound found mainly in Chinese herbal medicines such as astragalus and red clover. It is considered to be a typical phytooestrogen. In our previous experiments, it was found that formononetin has a two‐way regulatory effect on endothelial cells (ECs): low concentrations promote the proliferation of ECs and high concentrations have an inhibitory effect. To find a specific mechanism of action and provide a better clinical effect, we performed a structural transformation of formononetin and selected better medicinal properties for formononetin modifier J1 and J2 from a variety of modified constructs. The MTT assay measured the effects of drugs on human umbilical vein endothelial cell (HUVEC) activity. Scratch and transwell experiments validated the effects of the drugs on HUVEC migration and invasion. An in vivo assessment effect of the drugs on ovariectomized rats. Long‐chain non‐coding RNA for EWSAT1, which is abnormally highly expressed in HUVEC, was screened by gene chip, and the effect of the drug on its expression was detected by PCR after the drug was applied. The downstream factors and their pathways were analysed, and the changes in the protein levels after drug treatment were evaluated by Western blot. In conclusion, the mechanism of action of formononetin, J1 and J2 on ECs may be through EWSAT1‐TRAF6 and its downstream pathways.

## 1 INTRODUCTION

According to the literature, the consumption of vegetables and fruits is negatively correlated with cancer morbidity and mortality.^1^ It can also be said that eating more plant‐based foods has a certain effect on the incidence of cancer. Phytooestrogens are a class of non‐steroidal compounds found in plants.^2^ Red clover, also known as red axle, is a perennial herb of the genus Trifolium, which grows wildly in China's Xinjiang, Jilin, and Yunnan‐Guizhou plateaus and Hubei's mountainous areas.^3^ Research has shown that red clover contains a large number of isoflavones, which have anticancer and antitumour properties, regulate the balance of human hormones, improve osteoporosis, have antioxidant and free radical scavenging functions, etc Red clover is a natural medicine with great prospects.^4^, ^5^ Formononetin is a biologically active phytooestrogenic isoflavone extracted from red clover.

The phytooestrogens found in plant foods have non‐steroidal oestrogen‐like activity and are considered to be natural substitutes for oestrogen.^6^ The phytooestrogens can be divided into four categories: the isoflavones, alfalfa, lignans and coumarins.^7^ Among these categories, isoflavones (eg daidzein, genistein, calycosin and formononetin) dominate the research on phytooestrogens because they are the most biologically active ingredients in soya beans and they have been shown to significantly inhibit tumours.^8^, ^9^ Previous studies have shown that formononetin can be used as a pharmacological oestrogen analogue.^10^, ^11^ People have long recognized that tumour growth and metastasis require angiogenesis.^12^ Currently, HUVEC is considered to be a valuable in vitro angiogenesis model because they can form a capillary structure called a tube under appropriate stimulation.^13^ Studies have also shown that formononetin has antitumour effects in several types of cancer, such as breast cancer, osteosarcoma and ovarian cancer.^14^, ^15^ Our previous experimental results show that formononetin has a two‐way regulatory effect on HUVEC; that is, low concentrations promote proliferation of HUVEC, while high concentrations produce inhibition,^16^ but the specific mechanism remains to be understood. After conducting long‐term experiments, we found that the solubility of formononetin is low and that the concentration required inhibiting cells is too high. To compensate for these shortcomings, another laboratory that we worked with has structurally modified the drug, and in many modified species, J1 and J2 (purity > 98.0%) have been screened.

The growth of blood vessels, a process known as angiogenesis, is a complex and co‐ordinated process that involves multiple cellular cytokines and signal pathways.^13^ Insulin‐like growth factor‐1 (IGF‐1) is a new target to treat cardiovascular disease, effectively inhibiting EC apoptosis and promoting cell growth and migration through binding to the IGF‐1 receptor (IGF‐1R), which is expressed on the surface of vascular endothelial cells (VECs).^17^ Additionally, intercellular adhesion molecule 1 (ICAM‐1) is an Ig‐like cell adhesion molecule that is excreted by several types of cells, including leucocytes and ECs.^18^ ICAM‐1 is involved in polymorphonuclear leucocyte‐induced angiogenesis and is an early hallmark of the angiogenesis process. It endows the adhesive interaction between VECs and the extracellular matrix, hence promoting the growth of vascular endothelial cells.^2^, ^19^ These factors stimulate the proliferation, differentiation and metastasis of vascular endothelial cells; promote the formation of new blood vessels; provide sufficient oxygen and the nutrients necessary for tumour growth; provide a good growth environment for tumours; and accelerate tumour growth and proliferation. Therefore, inhibition of the formation of blood vessels has become a key step in the treatment of cancer.

It has recently been reported that long non‐coding RNAs (lncRNAs) are capable of regulating the proliferation, migration, invasion and chemoresistance of certain cells and are now considered to be novel regulators of gene expression.^20^, ^21^, ^22^, ^23^, ^24^, ^25^, ^26^ In addition, lncRNA is also involved in the process of cardiovascular development and pathophysiology.^27^

In this study, we initially screened several lncRNAs that were abnormally highly expressed in HUVEC but showed decreased gene expression levels after drug treatment. The difference in the expression of EWSAT1 was found to be more significant. Next, the downstream target was predicted to be TRAF6 with EWSAT1 as the upstream target. TRAF6 is involved in the downstream pathway TAK1‐C‐jun/IκBα. To explore the mechanism of this pathway and the role of the drugs, we performed the following research.

## 2 MATERIALS AND METHODS

### 2.1 Cell culture

HUVEC (The Chinese Academy of Sciences) was cultured in RPMI‐1640 medium (Invitrogen) containing 10% foetal bovine serum (FBS; Gibco) and cultured at 37°C, a 5% CO_2_ concentration and saturated humidity. The cells were well adhered and routinely digested according to the density and fusion of the cells, and the medium was replaced with phenol red‐free RPMI‐1640 medium (Gibco) (containing 10% foetal bovine serum solution treated with dextran) 4 days before the experiment. Then, the cells were allowed to grow for 4 days. In addition, the medium was changed to low‐serum RPMI‐1640 medium (containing 0.5% foetal bovine serum solution) for 24 hours before the cell experiments.

### 2.2 MTT assay

Formononetin (C_16_H_12_O_4_) (purity > 98.0%, Yuanye Biotechnology Co., Ltd.) and the J1 (C_24_H_24_O_9_) (purity > 98.0%) and J2 (C_28_H_34_N_2_O_7_) (purity > 98.0%) were dissolved in dimethyl sulphoxide (DMSO) to prepare J1 and J2 stock solutions, which was stored at 4°C for further use. Cell proliferation after treatment with J1 and J2 was tested by the MTT assay. Human umbilical vein endothelial cell was inoculated in 96‐well plates (5 × 10^3^ cells per well) for 12 hours and then exposed to various concentrations of J1 or J2 (0, 0.1, 0.5, 1, 2, 4, 8, 16, 32 μmol/L) (0 group added to DMSO). After 48 hours, the cells were incubated with 3‐[4,5‐dimethylthiazol‐2‐yl]‐2,5‐diphenyltetrazolium bromide (MTT) for 4 hours and then lysed in DMSO. Optical density (OD) values were measured at 490 nm using a plate reader (BioTek Instruments).

### 2.3 Wound healing assay

Cell migration ability was assessed with a wound healing assay. Equal numbers of cells were cultured in 6‐well plates until 95% confluence was reached. Wound gaps were created by scraping the cell sheets with a sterile 10‐µL pipette tip. The floating cells were removed by washing the wells with phosphate‐buffered saline (PBS). Changes in the scratched area were observed daily for 4 days by an inverted microscope (Leica). The wound width was measured to calculate the cell migration ability.

### 2.4 Plate colony formation experiment

Human umbilical vein endothelial cell was seeded at a low density in 6‐well plates (500 cells/well in triplicate) and was cultured for 2 weeks. Then, the cells were washed twice with PBS, fixed with 4% paraformaldehyde for 15 minutes and stained with a Gram staining solution for 20 minutes.

### 2.5 Transwell invasion assay

Cell invasion was assessed using a 24‐well Millicell suspension cell culture insert with an 8 µm polyethylene terephthalate (PET) membrane (Millipore) according to the manufacturer's instructions. Subsequently, 5 × 10^4^ cells from each group were suspended in 200 µL of serum‐free medium and inoculated into the upper chamber. Then, formononetin and J1 were added, and 500 µL of complete medium containing 10% FBS was added to the lower chamber. After incubation at 37°C for 48 hours, the non‐invasive cells were carefully removed from the upper surface of the filter. The invading cells in the lower chamber (below the surface of the filter) were fixed in 100% methanol, stained with 0.1 mg/mL crystal violet solution (Beyotime Biotechnology) and counted under a microscope. Five random fields of view were counted for each well, and the mean was determined.

### 2.6 Apoptosis staining

Cells were seeded in 6‐well plates (12 × 10^4^ cells/well) and cultured to confluence with different concentrations of formononetin (0, 16, 32 and 64 μmol/L) or J1 (0, 4, 8 and 16 μmol/L) (0 group added to DMSO) for 48 hours. The nuclear morphology of the apoptotic cells seeded in the 6‐well plates was observed using Hoechst 33258 stain. Briefly, cells were washed three times with PBS for 10 minutes, fixed in 4% paraformaldehyde for 20 minutes at 4°C, washed three times with PBS, stained with Hoechst 33258 (500 μL/well, Beyotime Institute of Biotechnology) stained for 5 minutes, washed again with PBS three times, covered with anti‐fade solution and then observed using the abovementioned fluorescence microscope (magnification, ×400).

### 2.7 Microarray of lncRNAs

Total RNA was extracted from HUVEC using the miRNeasy mini kit (Qiagen) according to the manufacturer's protocol. RNA amplification and labelling were performed using the Amino Allyl MessageAmp II aRNA Amplification Kit (Ambion). Labelled cDNA was subjected to hybridization using the Human lncRNA OneArray Plus microarray (Phalanx Biotech Group), followed by scanning using an Agilent scanner (Agilent Technologies). Agilent Feature Extraction software (version 11.0.1.1) was used to grid and extract the data.

### 2.8 Gene function analysis

The predicted target genes of differentially expressed lncRNAs were input into the Database for Annotation, Visualization and Integrated Discovery, which utilizes Gene Ontology (GO) analysis to analyse the molecular functions represented in the lncRNA profile (XuanC Bio). Furthermore, the potential functions of these differentially expressed lncRNAs in the pathways were determined by the Kyoto Encyclopedia of Genes and Genomes database.

### 2.9 Quantitative real‐time PCR (qRT‐PCR) assay

First, RNA was extracted from HUVEC using TRIzol (Gibco‐BRL). A total of 20 ng of RNA was used for reverse transcription, which was carried out using the Revert Aid First Strand cDNA Synthesis Kit (Fermentas, Life Sciences). The quantification of EWSAT1, TRAF6 and GAPDH was performed via qRT‐PCR. The primers used for qRT‐PCR were as follows: EWSAT1‐F: GTGTCTGGCAAGGAACACTA and EWSAT1‐R: GGTGGAGAAGAGGGACAATAAG, TRAF6‐F: TCATTATGATCTGGACTGCCCAAC and TRAF6‐R: TGCAAGTGTCGTGCCAAGTG, and GAPDH F: GCTACACTGAGGACCAGGTTGTC and GAPDH‐R: AGCCGTATTCATTGTCATACCAGG. Then, HUVEC was treated with the J1 (0, 4, 8 and 16 μmol/L) formononetin derivative. After 48 hours, the RNA was extracted and reverse‐transcribed into cDNA. The expression levels of EWSAT1 and TRAF6 in HUVEC treated with various concentrations of the J1 formononetin derivative were quantified by qRT‐PCR, and GAPDH was used as a housekeeping gene to calculate the relative expression levels of EWSAT1 and TRAF6.

### 2.10 Western blot analysis

Human umbilical vein endothelial cell was treated with the J1. After 48 hours, cell lysates were harvested, and the protein concentration was determined using a Bio‐Rad assay kit (Bio‐Rad Laboratories). Equal amounts of protein were separated by SDS‐PAGE and transferred to a 0.22‐μm polyvinylidene fluoride (PVDF) membrane (Bio‐Rad Laboratories). Then, the membrane was blocked with TBST (Tris‐buffered saline, pH 7.6, 0.05% Tween‐20) containing 5% skim milk powder for 2 hours. Membranes were then incubated overnight at 4°C with the indicated concentrations of the following primary antibodies: TRAF6, 1:1000 (Abcam); IGF‐1R, 1:1000 (Abcam); β‐actin, 1:500 (Zsgb Bio); p‐TAK1, 1:5000 (Abcam); TAK1, 1:5000 (Abcam); p‐IκBα, 1:3000 (Abcam); IκBα, 1:5000 (Abcam); p‐C‐jun, 1:5000 (Abcam); and C‐jun, 1:5000 (Abcam). After washing three times with TBST, the blot was incubated with a suitable secondary antibody conjugated to horseradish peroxidase at room temperature for 1 hour and then developed in an electrochemiluminescence Western blotting detection reagent (Beyotime). The expression level of the protein was compared to the expression level of the control based on the relative intensity of the bands.

### 2.11 Drug and animals

Formononetin (purity > 98.0%, Yuanye Biotechnology Co., Ltd.) was dissolved in DMSO to prepare a 160 mg/mL stock solution that was stored at 4°C for further use. Sprague Dawley rats (female, 6 weeks old, 200‐220 g) were supplied by Hunan SJA Laboratory Animal Co., Ltd. Animals were bred in a specific pathogen‐free room under a constant temperature of 21‐25°C and 50%‐60% relative humidity with a 12‐hour light/12‐hour dark cycle. All experimental procedures were performed in accordance with the guidelines of the Experimental Research Institute of Guilin Medical University.

### 2.12 Ovariectomy

For the establishment of ovariectomized rat models, 40 female SD rats were randomly divided into 4 groups: sham operation group (SHAM group, n = 10), ovariectomized group (OVX group, n = 10), 8 mg/kg/d formononetin group (n = 10) and 16 mg/kg/d formononetin group (n = 10). After being anaesthetized by intraperitoneal (i.p.) injection of 40 mg/kg pentobarbital sodium, both ovaries were removed from the SD rats to establish ovariectomized rat models. Five days after surgery, drug administration groups were given 8 mg/kg/d or 16 mg/kg/d formononetin, respectively, by gavage once a day for 40 days. The animals in the SHAM and OVX groups received saline. At the end of the administration, the uteri, thoracic aortas and breast tissues of the rats in the 4 groups were removed and reserved carefully for follow‐up analysis.

### 2.13 Quantitative real‐time PCR (qRT‐PCR) assay in vivo

For the analysis of IGF‐1R and ICAM‐1 mRNA transcription, the uteri from different groups were collected, and the total RNAs were extracted by TRIzol reagent (Invitrogen) according to the standard protocol. The expression levels of the transcripts encoding IGF‐1R and ICAM‐1 were quantified using a Quant One Step qRT‐PCR Kit (SYBR Green) (TIANGEN) and sampled by the CFX96 Touch™ Real‐Time System (Bio‐Rad). The specific primers used in qRT‐PCR for IGF‐1R, ICAM‐1 and β‐actin were IGF‐IR‐F: 5′‐AACATCCGGCGAGGCAATAA‐3′ and IGF‐IR‐R: 5′‐TTCACGTAGCCAGTCACCAC‐3′, ICAM‐1‐F: 5′‐GAGCGACATTGGGGAAGACA‐3′ and ICAM‐1‐R: 5′‐CACTCGCTCTGGGAACGAATA‐3′, β‐actin‐F: 5′‐TGTCACCAACTGGGACGATA‐3′ and β‐actin‐R: 5′‐ GGGGTGTTGAAGGTCTCAAA‐3′.

### 2.14 Western blot assay in vivo

For the analysis of IGF‐1R and ICAM‐1 protein expression, the uteri from different groups were collected, and the total protein was extracted by RIPA reagent (Solarbio) by adding 1% PMSF protease inhibitors (Solarbio) according to the standard protocol. Then, the mixtures were sonicated on ice with a Sonifier cell disruptor (75%, 5 minutes). After incubation for 30 min on ice, the mixtures were centrifuged at 16 099.2 *g* for 10 min at 4°C. The concentration of the supernatant was determined with a BCA protein assay kit. Ten micrograms of protein was separated by 10% or 8% SDS‐polyacrylamide gel, and then, the protein in the gel was transferred to the activated PVDF membrane. After sealing with 5% skim milk, the PVDF membranes were incubated with the corresponding IGF‐1R antibody (1:1000) (Abcam), ICAM‐1 antibody (1:1000) (Abcam) or β‐actin antibody (1:500) (Zsgb Bio) at 4°C overnight, according to the molecular weights of the different proteins. The next day, the membranes were washed with TBST three times and then incubated with anti‐rabbit IgG/HRP (1:2000) (Zsgb Bio) and goat antimouse IgG/HRP (1:2000) (Zsgb Bio) for 2 hours. Protein bands were visualized using electrochemiluminescence (ECL) Western blot detection reagents (Beyotime) under a ChemiDoc™ XRS (Bio‐Rad) system.

### 2.15 Immunohistochemistry

The uteri, thoracic aortas and breast tissues from the different groups were collected and fixed in 4% paraformaldehyde overnight, dehydrated using a series gradient of ethanol, carefully embedded in paraffin and sectioned into 5‐μm‐thick slices. After deparaffinization in xylene and hydration with a series gradient of ethanol, sections of the tissues were incubated with 3% H_2_O_2_ for 10 minutes, followed by three PBS washes. Antigen retrieval from the samples was conducted by microwave treatment in citrate buffer (pH 6.8). Then, sections were separately incubated with primary antibodies: anti‐IGF‐1R receptor antibody (1:200) (Abcam) and anti‐ICAM‐1 antibody (1:200) (Abcam) at a constant temperature of 4°C overnight. After washing three times with PBS, sections were probed with the corresponding secondary antibody using a PV‐9000 polymer detection kit (Zhongshan), and immunoreactivity was visualized using 3,3‐diaminobenzidine (DAB). After counterstaining with haematoxylin, sections were observed under a light microscope (Olympus).

### 2.16 Statistical analysis

All data are presented as the mean ± standard deviation (SD). Statistical significance was tested by two‐tailed Student's *t* test or one‐way ANOVA using SPSS 19.0 software. Statistical significance was set at *P* < .05, and extreme significance was set at *P* < .01. All statistical diagrams were performed using GraphPad Prism 5.0.

## 3 RESULTS

### 3.1 Inhibitory effects of different concentrations of formononetin and J1 and J2 on endothelial cells

**Figure 1:**
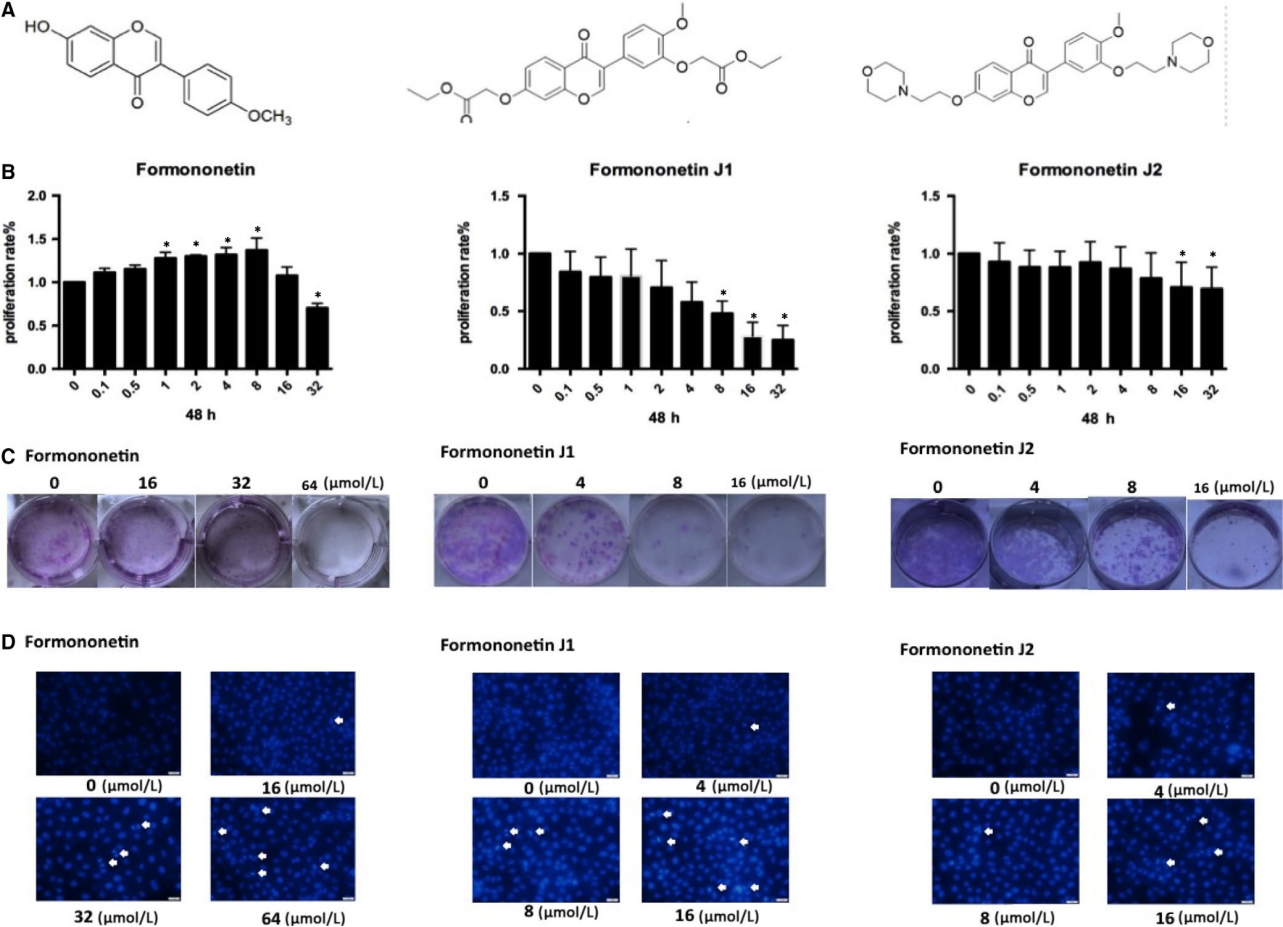
(A) Is the molecular structure of formononetin, J1 and J2, respectively. (B) HUVECs were treated with different concentrations of formononetin, J1 and J2 (0, 0.1, 0.5, 1, 2, 4, 8, 16, 32 μmol/L) for 48 h. The final concentrations of the compounds are shown. The cell proliferation rates were determined by the MTT assay (mean ± SD). *P < .05 vs control group (0 μmol/L). (C) Representative pictures of the number of HUVEC colonies. HUVECs were treated with formononetin (0, 16, 32 and 64 μmol/L) and J1 and J2 (0, 4, 8 and 16 μmol/L). (D) In the apoptosis staining experiment, HUVEC was treated with different concentrations of three drugs for 48 h, and more apoptotic bodies were observed in the high concentration group

Due to the solubility of formononetin, the concentration of the drug required to inhibit cells was too high, so modification of the formononetin structure was carried out to obtain two modified drugs, J1 and J2. Figure 1A shows the molecular structure of each drug. To elucidate the effects of these two plus formononetin on endothelial cells, MTT experiments were performed to examine the effects of these drugs on cells and to determine which J1 and J2 have better effects. As shown in Figure 1B, with increasing concentrations of formononetin and J1, an inhibitory effect was observed on HUVEC. However, the inhibition of J1 is stronger than that of J2. It can also be seen in Figure 1C,D that high concentrations of J1 and J2 have an inhibitory effect on HUVEC, and similarly, the inhibitory effect of J1 is significantly stronger than J2.

### 3.2 The J1 and J2 affected the invasion and migration of HUVEC

**Figure 2:**
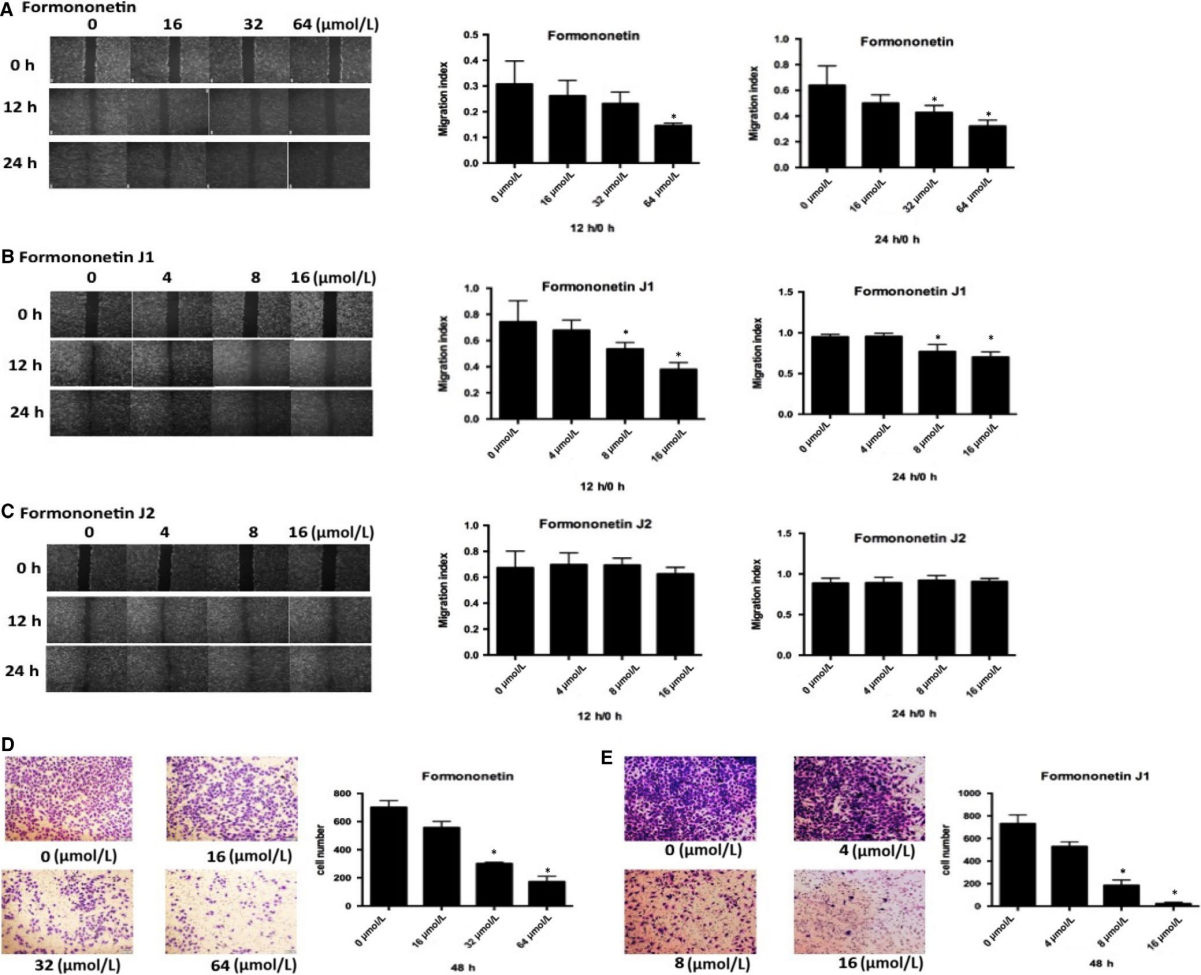
(A, B and C) Representative pictures and quantification of the migration index of HUVECs treated with different concentrations of formononetin (0, 16, 32 and 64 μmol/L) and J1 and J2 (0, 4, 8 and 16 μmol/L) for 12 h and 24 h. Each bar represents the mean ± SD of three independent experiments. *P < .05 vs control group (0 μmol/L). (D, E) Representative pictures and quantification of invaded cells, as analysed using the transwell matrix penetration assay. Each bar represents the mean ± SD of three independent experiments. *P < .05 vs control group (0 μmol/L)

We used several different concentrations of each J1 and J2 (0, 4, 8 and 16 μmol/L) and formononetin (0, 16, 32 and 64 μmol/L) to examine the effect of the drugs on the migratory ability of HUVEC. Figure 2A‐C shows the experimental results. The relatively high concentrations of J1 had a significant effect on HUVEC at 12 hours and 24 hours, while the effect of formononetin and J2 was not notable. The drug effect of J1 was significantly stronger than that of J2, so we used formononetin and J1 for the transwell experiment. Based on the results of the transwell experiment (Figure 2D,E), we concluded that as the concentrations of formononetin or J1 increase, fewer HUVEC pass through the basal lamina, and formononetin and J1 are more effective at higher concentrations. It can be concluded that the concentration required for the inhibitory effect from formononetin on HUVEC was significantly higher than that of J1, which also indicates that the inhibitory effect of J1 is better.

### 3.3 Effect of formononetin on ovariectomized rats

**Figure 3:**
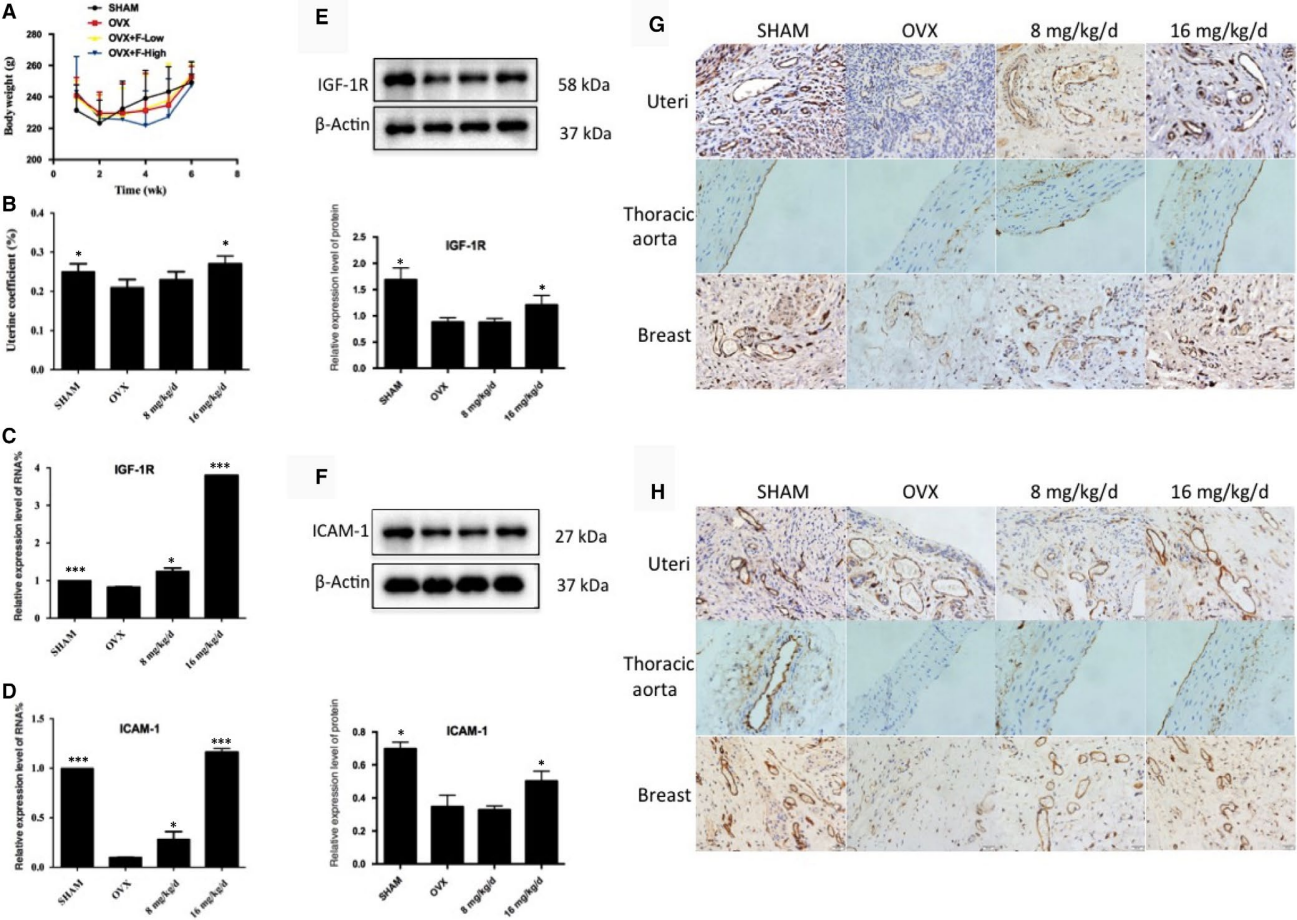
(A) Bodyweights of ovariectomized rats in during the treatments. (B) Uterine coefficient of ovariectomized rats in different groups, *P < .05, compared with OVX group. (C,D) The IGF‐1R and ICAM‐1 mRNA expression of uteri in different groups determined by qRT‐PCR. ***P < .001, compared with OVX group. (E,F) The IGF‐1R and ICAM‐1 protein expression of uteri in different groups determined by Western blot. *P < .05, **P < .01, compared with OVX group. (G,H) Effects of formononetin on the expression of IGF‐1R and ICAM‐1 in uteri, thoracic aortas and breast tissues of ovariectomized rats determined by immunocytochemistry method. Scale bar: 50 μm

The bodyweight of the rats slightly increased during the administration (Figure 3A). The uterine coefficient represents the relative weight of the uterus to the bodyweight, and the results showed that after 16 mg/kg/d administration, the uterine coefficient significantly increased compared with the OVX model group (Figure 3B), suggesting that 16 mg/kg/d group may promote uterine growth by oestrogen‐like effects. However, the effect of 8 mg/kg/d group is not good.

Next, to validate that formononetin possessed a protective effect against VECs in ovariectomized rats, we evaluated IGF‐1R and ICAM‐1 mRNA and protein expression levels in the uteri of different experimental groups. The results showed that after removing the ovaries of the rats to mimic early postmenopausal women, the OVX group exhibited lower IGF‐1R and ICAM‐1 mRNA and protein expression levels when compared with the SHAM group. However, after the administration of formononetin, 16 mg/kg/d group was remarkably up‐regulated the IGF‐1R and ICAM‐1 mRNA and protein expression levels. In the 8 mg/kg/d group, the level of mRNA was increased, but the protein level did not change (Figure 3C‐F). These results suggest that 16 mg/kg/d group, through its up‐regulation of angiogenesis‐associated factors (IGF‐1R and ICAM‐1), may promote growth and exert protective effects against VECs in uteri.

With the positive data supported above, we further demonstrated the IGF‐1R and ICAM‐1 protein expression in the uteri, thoracic aortas and breast tissues by immunocytochemistry assay. After administration of formononetin, the 8 mg/kg/d and 16 mg/kg/d groups exhibited higher expression levels of IGF‐1R and ICAM‐1 in the uterine cell vascular membrane compared with the OVX group (Figure 3G,H). The same trends were also observed in the thoracic aortas and breast tissues. Here, we concluded that formononetin up‐regulated the expression of IGF‐1R and ICAM‐1, thus achieving protective and angiogenic effects in the VECs of the uteri, thoracic aortas and breast tissues of ovariectomized rats.

Here, we also used the trend effects of J1 and J2 on rats for in vivo experiments. The trending effects of J1 and J2 in the in vivo experiments were not statistically significant.

### 3.4 EWSAT1‐TRAF6 participates in the influence of the HUVEC mechanism

**Figure 4:**
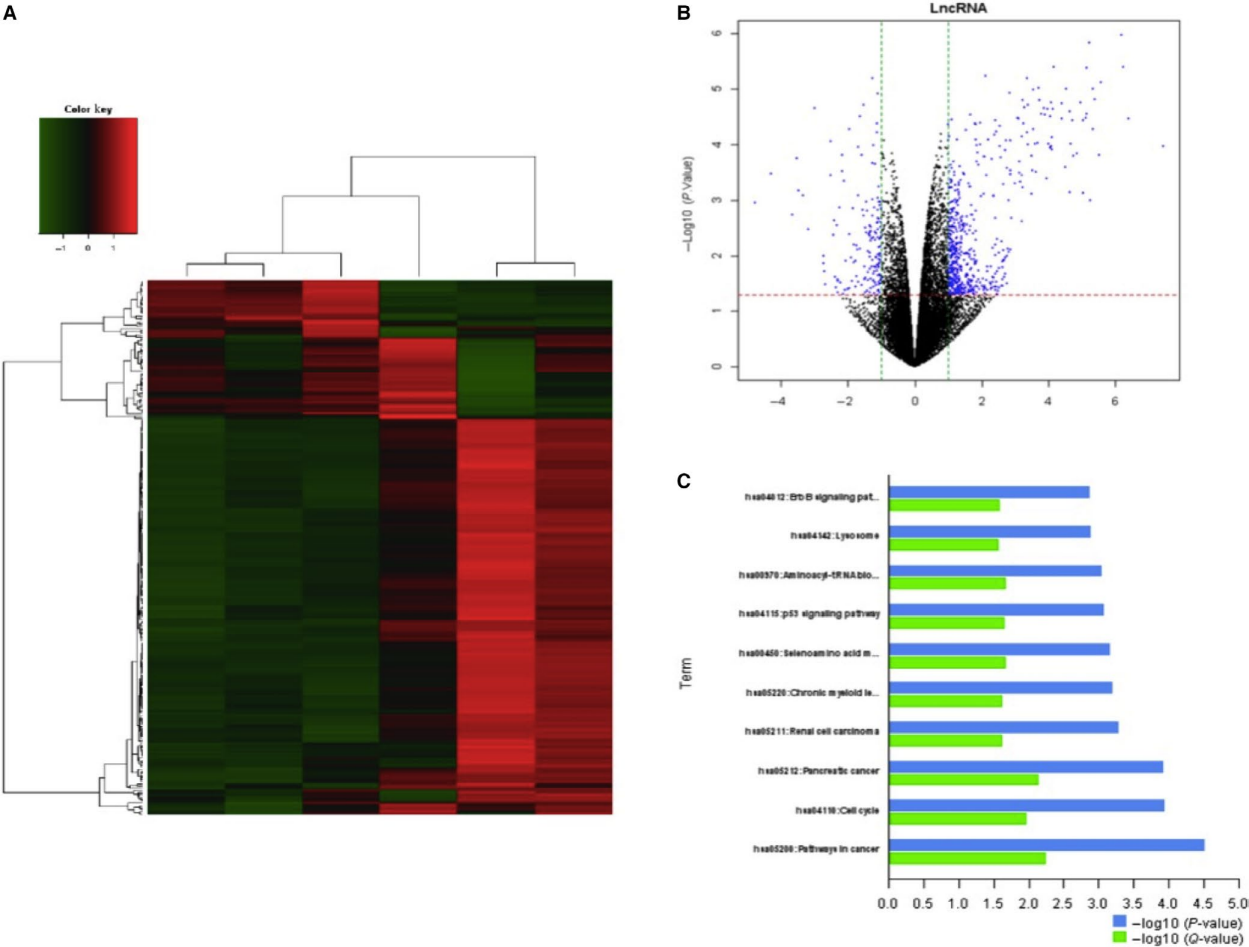
lncRNAs expression profile differences between the formononetin‐, J1‐ and J2‐treated groups compared to the control group. (A) The heat map, (B) the volcano plots, (C) the downstream pathway analysis

To explore the underlying mechanisms of formononetin, J1 and J2 regulation of endothelial cells, lncRNA levels were measured with microarrays after treatment with formononetin, J1 and J2 (Figure 4). J1 significantly down‐regulated the expression level of EWSAT1. Next, the downstream target of EWSAT1 was analysed, and it was determined that TRAF6 is a downstream target of EWSAT1.

### 3.5 Effect of J1 on the expression of EWSAT1 and its related downstream pathway factors

**Figure 5:**
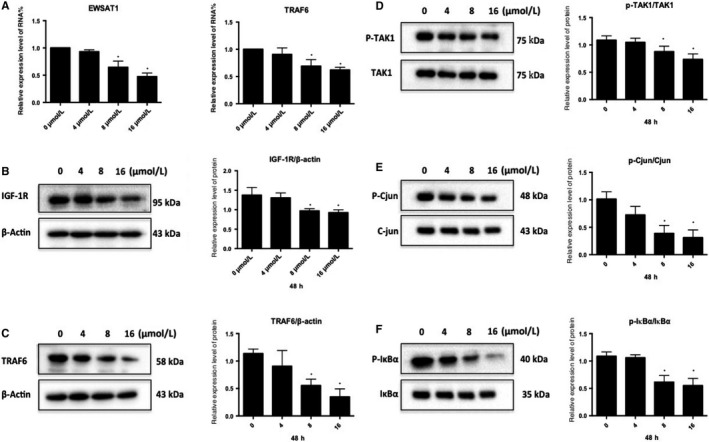
(A) HUVECs were treated with various concentrations of the J1 (0, 4, 8 and 16 μmol/L) for 48 h, and then, the EWSAT1 and TRAF6 mRNA expression levels were assessed by real‐time PCR (mean ± SD). The results are representative of three independent experiments performed in triplicate. *P < .05 vs control group (0 μmol/L). HUVECs were treated with various concentrations of the J1 (0, 4, 8 and 16 μmol/L) for 48 h, and then, the protein expression levels of IGF‐1R (B), TRAF6 (C), p‐TAK1 (D), p‐c‐Jun (E) and p‐IкBα (F) were determined by Western blot (mean ± SD). The results are representative of three independent experiments performed in triplicate. *P < .05 vs control group (0 μmol/L)

In this study (Figure 5A), after treating HUVEC with J1, the expression level of EWSAT1 in HUVEC was found to gradually decrease with an increasing concentration of J1. Moreover, the expression level of TRAF6 was also found to decrease accordingly. Simultaneously, the expression of TRAF6 and IGF‐1R and the phosphorylation of the related pathway proteins were also detected. As shown in Figure 5B‐F, the expression levels of TRAF6 and IGF‐1R gradually decreased with increasing J1 concentration, and the phosphorylation level of the related pathway protein factor TAK1 and its downstream pathway‐related protein also appeared to change. This indicates that J1 reduces the expression levels of TRAF6 and IGF‐1R, thereby affecting the phosphorylation of TAK1 and IGF‐1R and the downstream branches.

## 4 DISCUSSION

The proliferation of endothelial cells plays a key role in the process of angiogenesis, which is necessary for tumour growth and metastasis.^28^ Therefore, the endothelium is positioned as a barrier between the blood and blood vessels, and anticancer agents are damaging to the endothelial layer.^29^ Angiogenesis is thought to be a hallmark of cancer progression in solid tumours larger than 1‐2 mm.^3^, ^30^ Cancer cells grow rapidly due to the increased vascular supply, which maintains the delivery of oxygen and nutrients as well as the elimination of the waste generated by cells.^31^ Hypoxic and nutrient‐starved tumours secrete pro‐angiogenic factors that target specific receptors on the surface of vascular cells to promote neovascularization.^6^ Therefore, controlling the formation of blood vessels has become one of the primary tasks involved in treating tumours. Human umbilical vein endothelial cell is considered to be valuable in vitro angiogenesis model because they can form a capillary structure called a tube under appropriate stimulation.^13^ This study also selected the most experimental model objects of HUVEC.

Our results indicate that formononetin has a two‐way regulatory effect on endothelial cells, that is, a low concentration (>16 μmol/L) promotes the proliferation of HUVEC, and a high concentration (<16 μmol/L) inhibits HUVEC, but the specific mechanism is still unclear.^16^ In addition, long‐term experimental studies have found some shortcomings of formononetin, that is, the solubility of the drug is poor and the concentration required for the drug effect is large. Considering these problems, the drug was modified to obtain formononetin modifier, J1 and J2, which were selected from numerous modifier. However, the trend of J1 and J2 in vivo is not statistically significant.

Next, six samples of HUVEC were prepared, which were formononetin (0, 10 μmol/L), J1 (0, 10 μmol/L) and J2 (0, 10 μmol/L), and subjected to gene screening. Several lncRNAs were found to be highly expressed in HUVEC, and the fold change of EWSAT1 after treatment with J1 was significantly higher than that of the other two drugs after treatment with drugs. At the same time, based on the above results, J1 had better effects on HUVEC than formononetin and J2. Therefore, mechanism experiment was carried out using J1. After EWSAT1 was screened, its downstream target was analysed and predicted, and TRAF6 was found to be the downstream target of EWSAT1.

TRAF6 is a member of the TNF receptor–associated factor (TRAF) family.^32^ Recent studies have shown that inhibition of the TRAF6/NF‐κB/MMP2 pathway can affect endothelial cell invasion and migration.^33^ Studies have also shown that inhibition of the AT1R‐NFκB‐TRAF6‐mitogen‐activated protein kinase (MAPK) pathway affects endothelial cell proliferation and vascular regeneration.^34^ These results may indicate that TRAF6 also plays an important role in endothelial cell proliferation, invasion and migration. Upon stimulation, TRAF6 is recruited into the receptor complex and activated by the IL‐1 receptor‐associated kinase 1 (IRAK1), which binds to the TRAF domain of TRAF6.^35^ The IRAK1/TRAF6 complex is then excised from the receptor and binds to the membrane portion of TAK1 and the TAK1‐associated binding proteins TAB1 and TAB2.^35^ IRAK1 remains on the membrane and subsequently degrades, and then, the complex of TRAF6, TAK1, TAB1 and TAB2 enters the cytoplasm, forming a large complex with Ubc13 and Uev1A.^36^ TRAF6 acts as an E3 ubiquitin ligase with Ubc13/Uev1A, catalysing the formation of a K63‐linked polyubiquitin chain and then activating TAK1.^37^, ^38^, ^39^ TAK1 then phosphorylates and activates MAPK and IκB kinase (IKK).^40^

Vascular growth, a process called angiogenesis, is a complex and co‐ordinated process involving multiple cytokines and signalling pathways.^41^, ^42^ Insulin‐like growth factor‐1 (IGF‐1) is a novel target that inhibits endothelial cell apoptosis by binding to the IGF‐1 receptor (IGF‐1R) and promoting cell growth and migration. Recently, studies have shown that IGF‐1R can inhibit the proliferation of osteosarcoma cells through its downstream pathways, JNK and C‐jun N‐terminal kinase.^43^

In this study, after treatment of HUVEC with different concentrations of the formononetin derivative J1 for 48 hours, the expression levels of TRAF6, IGF‐1R and C‐jun and the phosphorylation levels of TAK1, IκBα and C‐jun were detected. The levels of phosphorylation change, and it was found that with increasing J1 concentration, the expression level of the TRAF6 protein gradually decreased, the phosphorylation level of the corresponding pathway proteins (including TAK1) changed accordingly, and the IGF‐1R protein level also decreased. Correspondingly, the phosphorylation level of C‐jun downstream was also reduced accordingly.

In summary, the above experimental results show that formononetin and J1 and J2 have the ability to inhibit endothelial cell proliferation, invasion and migration. It was found that J1 is superior to formononetin and J2. In addition, the selected lncRNA (EWSAT1) and its downstream targets were analysed, and the corresponding downstream pathways were found. This study confirmed that the formononetin derivative J1 regulates the expression of TRAF6 and its downstream TAK1‐ and IκBα/C‐jun‐related factors by mediating the expression of EWSAT1, and the IGF‐1R‐C‐jun pathway also has an effect on ECs. These advances in the understanding of the drug action of formononetin and formononetin modifier and their inhibition of the angiogenic processes necessary for tumour growth and metastasis may provide opportunities for their clinical application in tumour therapy.

## CONFLICT OF INTEREST

The authors declare that the research was conducted in the absence of any commercial or financial relationships that could be construed as a potential conflict of interest.

## AUTHOR CONTRIBUTIONS

Zhaoquan Huang and Jian Chen conceived and designed the study. Xin Li conducted an experiment. Chen Huang, Chengliang Sui and Guangying Qi analysed the data. Xin Li, Chunmei Liang and Qian Yao Ren drafted and revised the manuscript. All authors read and approved the final manuscript.

## Data Availability

Data available on request from the authors.
